# Deletion of the African Swine Fever Virus Gen *I196L* in the Georgia2010 Isolate Genome Does Not Affect Virus Replication or Virulence in Domestic Pigs

**DOI:** 10.3390/v17050603

**Published:** 2025-04-23

**Authors:** Elizabeth Ramirez-Medina, Paul A. Azzinaro, Alyssa Valladares, Ediane Silva, Leeanna Burton, Leandro Sastre, Vivian O’Donnell, James J. Zhu, Douglas P. Gladue, Manuel V. Borca

**Affiliations:** 1 Foreign Animal Disease Research Unit, Plum Island Animal Disease Center (PIADC), Agricultural Research Service, U.S. Department of Agriculture, P.O. Box 848, Greenport, NY 11944, USA; paul.azzinaro@usda.gov (P.A.A.); alyssa.valladares@usda.gov (A.V.); ediane.silva@usda.gov (E.S.); leeanna.burton@usda.gov (L.B.); leandro.sastre@usda.gov (L.S.); james.zhu@usda.gov (J.J.Z.); 2 Foreign Animal Disease Research Unit, National Bio and Agro-Defense Facility, Agricultural Research Service, U.S. Department of Agriculture, Manhattan, KS 66502, USA; 3 Oak Ridge Institute for Science and Education (ORISE), Oak Ridge, TN 37830, USA; 4 U.S. Department of Agriculture, APHIS, Plum Island Animal Disease Center (PIADC), P.O. Box 848, Greenport, NY 11944, USA; vivian.odonnell@usda.gov

**Keywords:** ASFV, ASF, African swine fever virus, recombinant virus, ASFV virulence, ASFV *I196L* gene

## Abstract

African swine fever (ASF) is a lethal disease of domestic pigs that is currently challenging swine production in large areas of Eurasia and the Caribbean. The causative agent, ASF virus (ASFV), is a large, double-stranded, and structurally complex virus. The ASFV genome encodes for more than 160 proteins; however, the functions of most of them are still in the process of being characterized. Recently, ASFV gene *I196L* has been reported as being critically involved in disease production in domestic pigs. We report here that a recombinant virus derived from the Georgia 2010 isolate (ASFV-G) lacking the *I196L* gene, ASFV-G-∆I196L, had the same ability to replicate in primary cultures of swine macrophage and, when experimentally inoculated in pigs, produced a fatal form of the disease similar to that caused by the parental virulent ASFV-G. Therefore, deletion of the *I196L* gene does not significantly affect virus replication and virulence in domestic pigs of the ASFV Georgia 2010 isolate.

## 1. Introduction

African swine fever virus (ASFV) is the etiological agent of a contagious disease, African swine fever (ASF), of domestic and wild swine, which can present in a variety of clinical forms depending on the virus strain involved [1,2]. Since 2007, ASF has been widely distributed across Asia and Europe and has recently been detected in the Dominican Republic and Haiti [3]. In the last fifteen years, ASF has significantly affected pig production and is causing food shortages worldwide.

ASFV is a large and structurally complex double-enveloped virus. The most internal membrane wraps a protein capsid, which harbors a large (180–190 kilobase pairs) double-stranded DNA genome, which encodes more than 150 genes [2]. The function of many of these genes has not been experimentally characterized, so their function remains unknown. In particular, for important processes such as virus replication and virulence in domestic swine. Understanding the function of those genes played a critical role in the identification of ASFV genes involved in virus virulence in domestic pigs, leading to the rational development of live attenuated ASFV vaccine candidates [4,5,6,7,8,9,10,11,12].

Recently, it has been reported, as a preprint [13], that the ASFV gene *I196L*, a late-expressed gene, was involved in the processes of virus replication in swine macrophages and, importantly, virus virulence. Two recombinant viruses (SY18ΔI196L-1 and 2) produced by the deletion of the *I196L* gene in the genome of the highly virulent field strain SY18 have abolished their virulence when inoculated in domestic pigs. Animals intramuscularly (IM) inoculated with 10^6^ HAD_50_ did not present any clinical signs of ASF and, importantly, were protected against the challenge with the virulent parental strain SY18. Therefore, SY18ΔI196L-1 and 2 appear as potential experimental live attenuated vaccine strains.

Here, we report that a recombinant virus harboring a deletion of the *I196L* gene from the genome of the highly virulent Georgia2010 isolate (ASFV-G), ASFV-G-∆I196L, presents similar replication kinetics to that of the parental ASFV-G in primary swine macrophage cultures. Importantly, animals IM inoculated with 10^2^ HAD_50_ of ASFV-G-∆I196L present an almost indistinguishable virulent phenotype from that shown by ASFV-G. Therefore, in the conditions evaluated in this report, it does not appear the *I196L* gene plays any important role in the replication nor in the process of disease production in domestic pigs of the ASFV-G isolate. The potential causes for the discrepancies between data reported here and those presented by Fan et al. [13] are further discussed.

## 2. Materials and Methods

### 2.1. Viruses and Cells

Primary cultures of swine macrophages were produced as previously described and plated at a density of 5 × 10^6^ cells per mL [4,5,6,7]. The ASFV Georgia2010 (ASFV-G) is a highly virulent field strain kindly provided by Dr. Nino Vepkhvadze, from the Laboratory of the Ministry of Agriculture (LMA) in Tbilisi, Republic of Georgia [4,5,6,7]. Evaluation of virus replication kinetics was performed by running comparative growth curves between ASFV-G-∆I196L and the parental ASFV-G in primary swine macrophage cultures with a multiplicity of infection (MOI) of 0.01 HAD_50_ [5,6,7]. Samples were taken at different times post-infection (pi) and frozen at ≤−70 °C. Cell lysates were titrated using primary swine macrophages in 96-well plates. Presence of infected cells was detected by hemadsorption (HA), and virus titers were calculated as previously described [5,6,7].

### 2.2. Development of the ASFV I196L Gene Deletion Mutant

ASFV-G-∆I196L was developed via genetic manipulation of the ASFV-G strain by deleting the ASFV gene *I196L*. Genetic modification was produced by homologous recombination using a recombination transfer vector (named p72mCherryΔI196L) as previously described [5]. The recombination transfer vector p72mCherryΔI196L harbors the ASFV-G genomic regions flanking the *I196L* gene. The left flanking arm covers approximately 1000 base pairs to the left of nucleotide position 176,206, and the right arm also covers approximately 1000 base pairs to the right of nucleotide position 176,607. In addition, the recombinant vector also harbors the reporter gene cassette with the mCherry fluorescent protein (mCherry) gene under the control of the ASFV p72 late gene promoter [5]. p72mCherryΔI196L was developed by DNA synthesis (Epoch Life Sciences, Sugar Land, TX, USA). Recombinant ASFV-G-∆I196L harbors a partial deletion of the *I196L* gene (between nucleotide positions 176,206 and 176,607). ASFV-G-∆I196L was purified by limiting dilution based on mCherry activity detection. The full-length genome of ASFV-G-∆I196L was sequenced using next-generation sequencing (NGS) following exactly the procedures previously described [5].

### 2.3. Evaluation of ASFV-G-∆I196L Virulence in Domestic Pigs

The virulence of ASFV-G-∆I196L was evaluated in 35–40 kg commercial breed swine. Groups of pigs (*n* = 5) were intramuscularly (IM) inoculated with 10^2^ HAD_50_ of either ASFV-G-∆I196L or the virulent parental virus ASFV-G. The presence of clinical signs associated with ASF (such as anorexia, depression, fever, purple skin discoloration, staggering gait, diarrhea, and cough) as well as body temperature values were recorded daily throughout the experiment. Experiments with pigs were performed under biosafety level 3 conditions in the animal facilities at Plum Island Animal Disease Center, following a strict protocol approved by the Institutional Animal Care and Use Committee (225.07-14-R_090716, approved on 14 July 2022).

## 3. Results and Discussion

### 3.1. Structural Analysis of the ASFV I196L Gene

**Figure 1:**
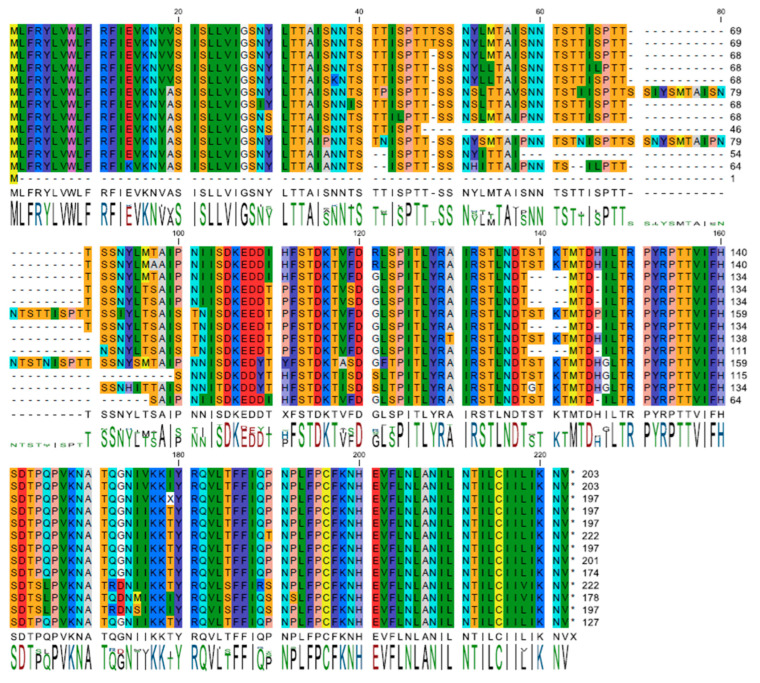
Unique sequence alignment of *I196L*. A dataset comprising translated, non-redundant I196L protein sequences was aligned using CLC software (version 23.0.2). The residues are represented in color according to the RasMol scheme. Bars below show conservation as compared to consensus. It is observed that the consensus is significantly lower within the direct repeat motif compared to other regions of the protein, whereas the C-terminal sequence exhibits nearly complete conservation.

ASFV gene *I196L* presents no significant amino acid sequence or structural similarities with other known proteins [14,15]. An analysis of a previously established database containing 220 annotated ASFV genomes led to the identification of a subset comprising 13 non-redundant sequences for I196L [16]. This collection of unique sequences was utilized to create an alignment and a consensus sequence (Figure 1) for the I196L protein. The consensus was determined using a simple majority approach. In the sole instance where no consensus was reached, residue 111 in Figure 1, histidine was chosen over proline for the consensus sequence to facilitate further modeling. Analysis of the sequence conservation appears highest in both the N-terminal (consensus residues 1–27) and C-terminal (consensus residues 164–196) regions and lowest in the repeated motif (consensus residues 28–84) that is more prone to insertions and deletions.

**Figure 2:**
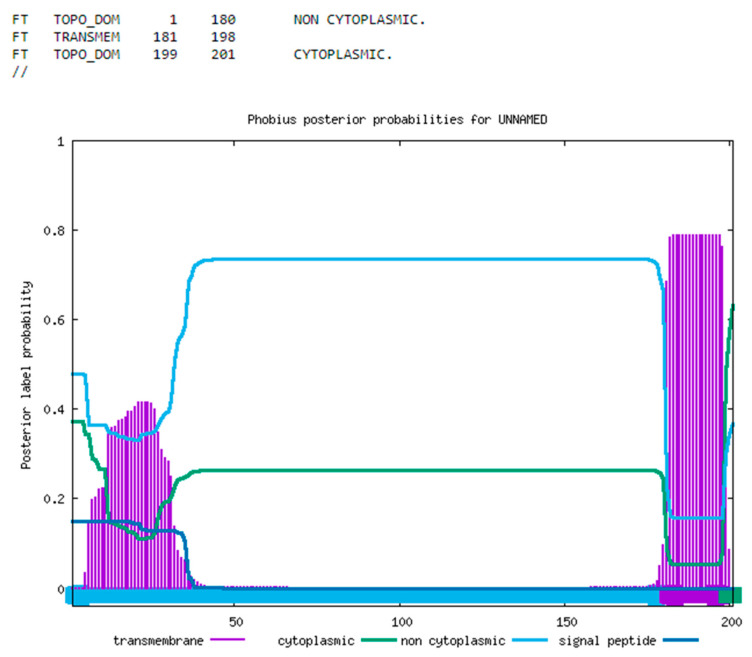
Subcellular location of I196L. The prediction of the consensus sequence for the I196L gene by Phobius suggests that the majority of the protein is localized outside the cytoplasm and highlights the presence of a transmembrane domain within the highly conserved C-terminal region.

The protein sequence was analyzed for known signal peptides utilizing Phobius (Figure 2), revealing the presence of a C-terminal transmembrane domain, while the remainder of the protein appears to be non-cytoplasmic [17]. Both nucleotide and protein BLAST (version 2.16.0) analyses failed to identify any homologous sequences beyond those associated with ASFV, and the examination of the protein sequence did not reveal any recognized protein families or domains, aside from low complexity regions that are likely to enhance structural fluidity, especially in disordered segments [18]. It is conceivable that these disordered regions may adopt stable conformations when interacting with partner molecules. In the absence of known interactors, these regions are likely to remain flexible, exhibiting high solvent accessibility and undergoing regular shape changes in response to energy fluctuations.

**Figure 3:**
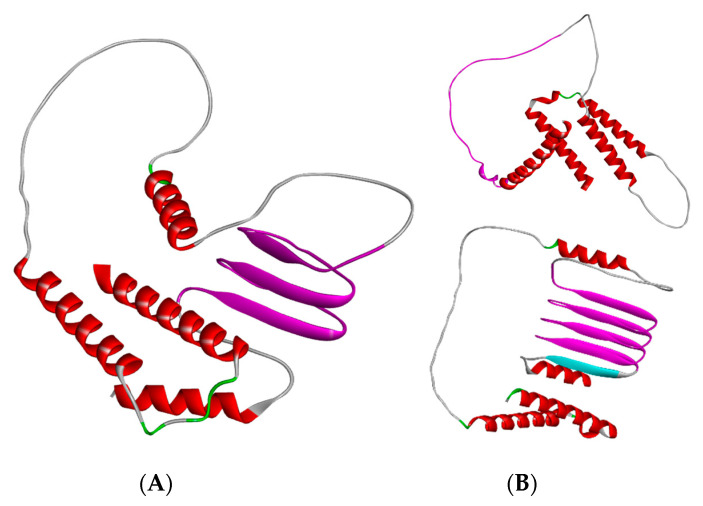
Structural models of *I196L*. (**A**) The AlphaFold3 model representing the consensus sequence for *I196L* exhibits certain variations when compared to the previously published AlphaFold2 model. The repeated motif is highlighted in magenta, while the remainder of the model is color-coded according to its secondary structure. Regions identified as unstructured display low confidence levels and are likely to possess a dynamic conformation. (**B**) The AlphaFold3 models for representative protein sequences feature either 2 (top) or 4 (bottom) instances of the tandem repeat sequence.

De novo structural modeling was previously conducted utilizing AlphaFold version 2.1.0, and new models have been generated with AlphaFold version 3.0.0 (Figure 3) [19,20]. Although proteins that contain transmembrane domains are particularly challenging to model, certain patterns can be observed. Various sequences exhibit duplications or deletions of a linear segment comprising approximately 22 amino acids, which is anticipated to form a beta sheet structure. The consensus sequence contains three occurrences of this unannotated motif. In contrast, other sequence variants possess only two occurrences, which fail to consistently produce the beta sheet structure, whereas variants with four repeats demonstrate a higher confidence score for the beta sheet formation (Figure 3B). This sequence appears to represent a tandem repeat with no reverse complement present elsewhere in the genome.

### 3.2. Development of the Recombinant ASFV-G-ΔI196L

**Figure 4:**
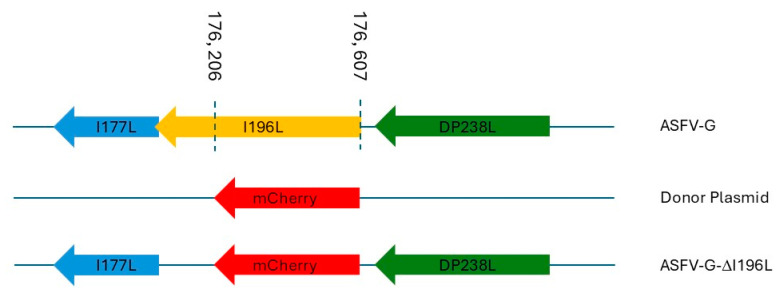
Schematic for the development of ASFV-G-∆I196L. The recombinant vector, containing the mCherry reporter gene under the ASFV p72 promoter activity, and the gene positions are shown. The nucleotide positions of the area that was deleted in the ASFV-G genome are indicated by the dashed lines. The resulting ASFV-G-∆I196L virus with the cassette inserted is shown on the bottom.

Deletion of the *I196L* gene in the Chinese field isolate SY18 produced a decrease in virus replication in swine macrophages and attenuation of virus virulence when inoculated in pigs [13]. To understand the potential role of the *I196L* gene in these processes regarding the virulent field isolate ASFV Georgia 2010 isolate (ASFV-G), a recombinant virus harboring the partial deletion of the *I196L* gene (ASFV-G-∆I196L) was developed using ASFV-G as the parental virus. The *I196L* gene, spanning between nucleotide positions 175,999 and 176,607, was partially deleted between nucleotide positions 176,206 and 176,607. The deleted area of the *I196L* gene was replaced by the 1226 bp p72mCherry cassette [5]. (Figure 4). The design of the construct was specifically focused on not altering the transcription of the closely adjacent *I177L* gene, which has been well characterized as being a powerful ASFV genetic virulent factor [5]. The stock of the recombinant ASFV-G-∆I196L was purified by limiting dilution using primary swine macrophage cell cultures as cell–substrate.

The full genome sequence of the recombinant ASFV-G-∆I196L was assessed by NGS using an Illumina NextSeq^®^ 500. Results of the analysis demonstrated a deletion of an area of 401 nucleotides inside the *I196L* gene and the insertion of 1226 nucleotides corresponding to the p72-mCherry cassette sequence. There were no unwanted genomic modifications detected in the stock of ASFV-G-∆I196L, and the NGS data showed no evidence of a potential contamination of the parental ASFV-G genome into the ASFV-G-∆I196L stock.

### 3.3. Replication of ASFV-G-∆I196L in Primary Swine Macrophages

**Figure 5:**
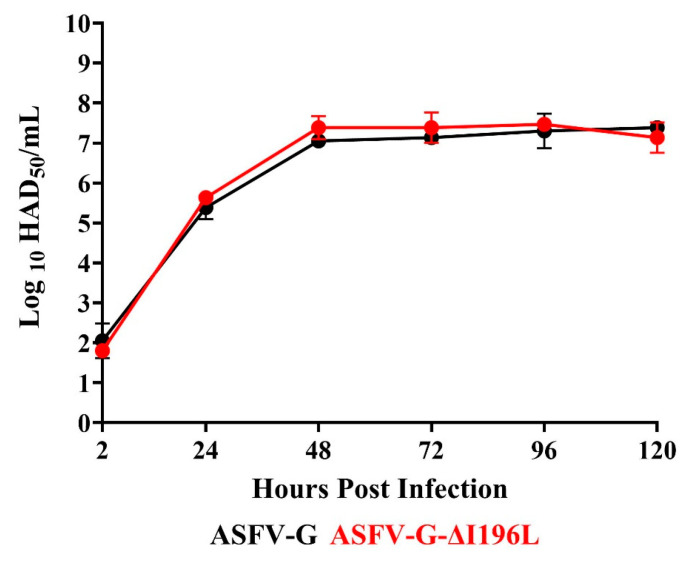
In vitro growth kinetics in primary swine macrophage cell cultures for ASFV-G-∆I196L and parental ASFV-G (MOI = 0.01). Data represents means and standard deviations of two replicas. Sensitivity using this methodology for detecting virus is ≥log10^1.8^ HAD_50_/mL.

To assess the potential role of the *I196L* gene in virus replication in swine macrophage cell cultures, the replication kinetics of ASFV-G-∆I196L were comparatively evaluated with those of the parental ASFV-G. A multistep growth curve was implemented, infecting swine macrophage cultures with an MOI of 0.01 with either ASFV-G-∆I196L or ASFV-G, and virus yields were assessed at 2, 24, 48, 72, 96, and 120 h pi. Results showed that the growth kinetics of ASFV-G-∆I196L and that of the parental ASFV-G practically overlap each other, suggesting that the deletion of the *I196L* gene from the genome of the ASFV-G does not affect the ability of the virus to replicate in swine macrophage cultures (Figure 5). This result differs from that reported by Fan et al. [13], where, under similar experimental conditions to those described here, two recombinant viruses derived from the virulent field strain SY18 harboring full-length deletions of the *I196L* gene showed a statistically significant decrease in virus yield in primary swine macrophage cultures.

### 3.4. Assessment of ASFV-G-∆I196L Virulence in Swine

To assess the potential role of the *I196L* gene in the virulence of the parental ASFV-G isolate, a group of five 35–40 kg pigs was IM inoculated with 10^2^ HAD_50_ of the recombinant ASFV-G-∆I196L. A control group of pigs (*n* = 4) with the same characteristics as those receiving the recombinant virus was inoculated with the virulent parental ASFV-G isolate using the same dose and route of inoculation. The presence of ASF-associated clinical signs was observed daily in both groups of animals.

**Figure 6:**
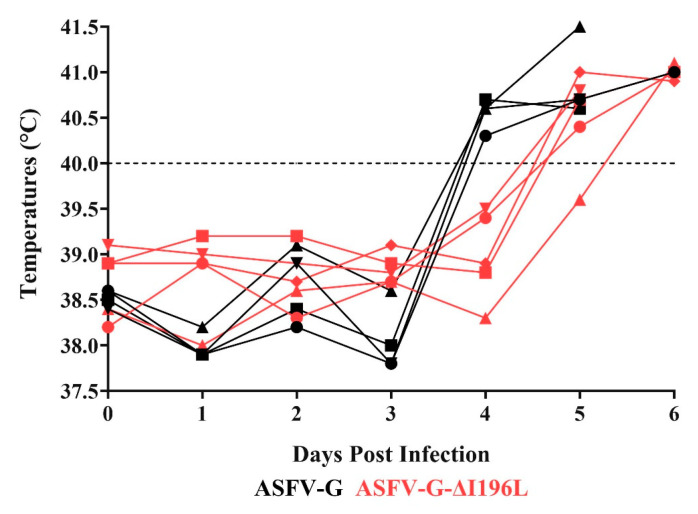
Evolution of body temperature in animals IM infected with 10^2^ HAD_50_ of either ASFV-G-∆196L or parental ASFV-G.

**Figure 7:**
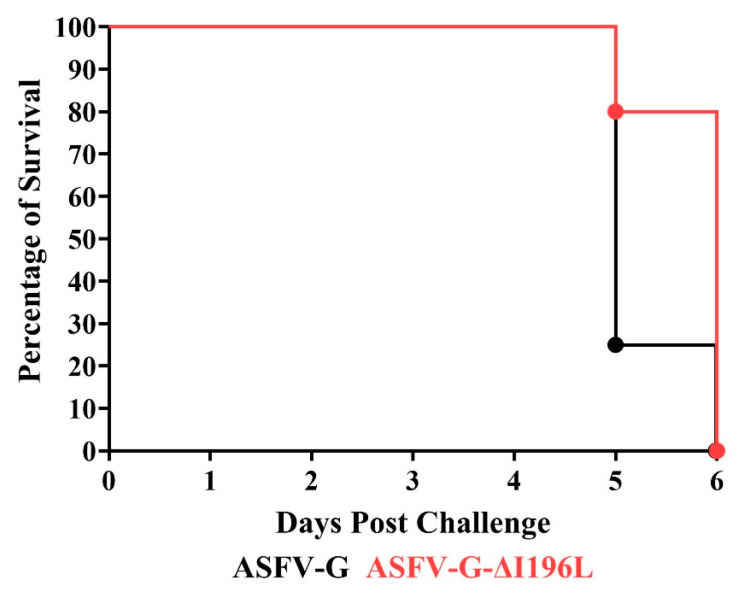
Evolution of mortality in animals IM infected with 10^2^ HAD_50_ of either ASFV-G-∆I196L or parental virulent ASFV-G.

As expected, all animals infected with the virulent parental ASFV-G presented a sudden increase in body temperature (>40 °C) on day 4 pi, progressing to full clinical disease (Figure 6 and Figure 7), with all pigs being euthanized on days 5–6 pi.

Animals inoculated with the recombinant ASFV-G-∆I196L also showed development of a lethal form of the disease, similar to that observed in the animals inoculated with the parental ASFV-G. All pigs in this group, except one, presented an increased body temperature over 40 °C on day 5 post infection, in all of them evolving to a more severe form of clinical disease, with all pigs euthanized between days 5 and 6 pi (Figure 6 and Figure 7). Therefore, the deletion of the *I196L* gene from the genome of the virulent ASFV-G isolate does not affect the level of virus virulence when tested in experimentally infected domestic swine under the experimental conditions described here. Again, these results have a striking contrast to those described by Fan et al. [13]. Those authors reported that doses as high as 10^6^ HAD_50_ of a recombinant virus harboring full-length deletion of the *I196L* gene in the virulent field strain SY18 resulted in a completely attenuated phenotype when inoculated in domestic pigs under similar experimental conditions as those reported here.

**Figure 8:**
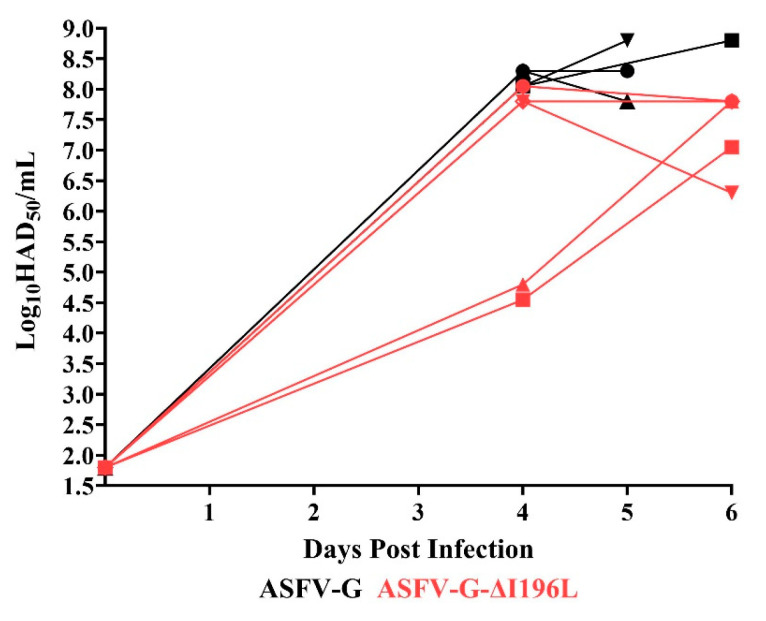
Viremia titers detected in pigs IM inoculated with 10^2^ HAD_50_ of either ASFV-G-∆ I196L or ASFV-G. Each symbol represents individual viremia titers in each animal in the groups. Sensitivity of virus detection: ≥log10 ^1.8^ HAD_50_/mL.

The replication of the recombinant ASFV-G-∆I196L in the infected pigs was assessed by analyzing the viremia titers and by comparing those to the viremia levels detected in animals inoculated with the parental virulent ASFV-G (Figure 8). Animals IM inoculated with ASFV-G presented high viremia titers (approximately 10^8^ HAD_50_/mL) by day 4 pi, maintaining similar values until days 5–6 pi, when all animals needed to be euthanized due to the severity of the clinical signs. Similarly, animals inoculated with the recombinant ASFV-G-∆I196L showed viremia titers ranging between 10^4.5^ and 10^8^ HAD_50_/mL by day 4 pi, evolving their viremia titers to 10^6^–10^7.5^ HAD_50_/mL by day 5–6 pi, when all of them had to be euthanized.

It is important to mention that full-length genomic sequences of viruses obtained in blood samples taken from the five animals inoculated with ASFV-G-∆I196L confirmed that the virus present in each of the samples sequenced by NGS was ASFV-G-∆I196L. These results confirm that the clinical disease present in those animals was in fact produced by ASFV-G-∆I196L and not by potential residual contamination from the parental ASFV-G remaining after the ASFV-G-∆I196L purification process. Therefore, ASFV-G-∆I196L produces a lytic systemic infection in experimentally infected animals similar to that present in animals inoculated with the parental ASFV-G.

Results reported here clearly show that the deletion of the *I196L* gene from the genome of the highly virulent isolate Georgia 2010 does not affect basic ASFV functions, such as virus replication in macrophages (both in cell cultures and experimentally infected pigs) and the virulence in domestic pigs of ASFV strain Georgia. As mentioned, when describing the obtained results, it is important to mention that these results oppose those reported by Fan et al. [13] when deleting the *I196L* gene in the genome of the virulent field strain SY18. Isolate SY18 is a derivative of the ASFV-G, and, therefore, comparison of the full-length genome sequences demonstrates a very high homology (99.98% or 25 nucleotide deletions/insertions and 4 substitutions). Furthermore, comparison of the amino acid sequence encoded by the *I196L* gene in ASFV-G and SY18 showed a 100% homology. A possible explanation of the phenotype differences between viruses lacking *I196L* in virulent field strains ASFV-G and SY18 is that the *I196L* gene may constitute another example of ASFV genes whose deletion produces different phenotypes depending on the genetic background (different virus isolates) where the deletion is performed, as was reported for genes such as *B119L* [7,21], *EP402R* [22,23], *DP96R* [24,25,26], *DP71L* [25,26], *B119L*/*DP96R* [4,21], *TK* [27,28], *DP148R* [29,30], and *I267L* [31,32].

An alternative cause for these discrepancies could be differences between the design of the deletion of the *I196L* gene in the ASFV-G-∆I196L presented here and those in the recombinant viruses reported by Fan et al. [13]. In the ASFV-G-∆I196L, there is a partial deletion of the *I196L* gene comprising the 5′ end of the *I196L* gene until ASFV nucleotide position 176,206, deleting the first 133 residues of the *I196L* gene. This construct preserves the integrity of the *I177L* gene and its promoter, located 1206 nucleotides downstream adjacently to the left of the closer deletion end of the *I196L* gene (ASFV nucleotide position 176,206). Conversely, the two recombinant viruses reported by Fan et al., SY18-∆I196L-1 and SY18-∆I196L-2, present either a complete deletion of the *I177L* promoter or a deletion of that promoter along with the first 8 nucleotides at the open reading frame of the *I177L* gene, respectively [13]. Therefore, it is possible that the reduced ability of replication in swine macrophages and, particularly, the attenuated phenotype of SY18-∆I196L-1 and SY18-∆I196L-2 could be due to failure in the expression of the *I177L* gene rather than the deletion of the *I196L* gene itself. In fact, the reduced ability of replication in swine macrophages as well as the level of attenuation in domestic pigs described for SY18-∆I196L-1 and SY18-∆I196L-2 [13] are quite comparable to those reported for ASFV-G-∆I177L [5]. Therefore, we believe deletion of the *I196L* gene does not affect virus replication in swine macrophages nor is it involved in the process of virulence during the ASFV infection in domestic pigs.

## Data Availability

All data are included in the manuscript.

## References

[1] Gonzales W., Moreno C., Duran U., Henao N., Bencosme M., Lora P., Reyes R., Nunez R., De Gracia A., Perez A.M. (2021). African swine fever in the Dominican Republic. Transbound. Emerg. Dis..

[2] Tulman E.R., Delhon G.A., Ku B.K., Rock D.L., Van Etten J.L. (2009). African Swine Fever Virus. Lesser Known Large dsDNA Viruses.

[3] Ramirez-Medina E., O’Donnell V., Silva E., Espinoza N., Velazquez-Salinas L., Moran K., Daite D.A., Barrette R., Fabuary B., Holland R. (2022). Experimental Infection of Domestic Pigs with an African Swine Fever Virus Field Strain Isolated in 2021 from the Dominican Republic. Viruses.

[4] O’Donnell V., Risatti G.R., Holinka L.G., Krug P.W., Carlson J., Velazquez-Salinas L., Azzinaro P.A., Gladue D.P., Borca M.V. (2017). Simultaneous deletion of the 9GL and UK genes from the African swine fever virus Georgia 2007 isolate offers increased safety and protection against homologous challenge. J. Virol..

[5] Borca M.V., Ramirez-Medina E., Silva E., Vuono E., Rai A., Pruitt S., Holinka L.G., Velazquez-Salinas L., Zhu J., Gladue D.P. (2020). Development of a Highly Effective African Swine Fever Virus Vaccine by Deletion of the I177L Gene Results in Sterile Immunity against the Current Epidemic Eurasia Strain. J. Virol..

[6] Gladue D.P., Ramirez-Medina E., Vuono E., Silva E., Rai A., Pruitt S., Espinoza N., Velazquez-Salinas L., Borca M.V. (2021). Deletion of the A137R Gene from the Pandemic Strain of African Swine Fever Virus Attenuates the Strain and Offers Protection against the Virulent Pandemic Virus. J. Virol..

[7] O’Donnell V., Holinka L.G., Krug P.W., Gladue D.P., Carlson J., Sanford B., Alfano M., Kramer E., Lu Z., Arzt J. (2015). African swine fever virus Georgia 2007 with a deletion of virulence-associated gene 9GL (B119L), when administered at low doses, leads to virus attenuation in swine and induces an effective protection against homologous challenge. J. Virol..

[8] Monteagudo P.L., Lacasta A., Lopez E., Bosch L., Collado J., Pina-Pedrero S., Correa-Fiz F., Accensi F., Navas M.J., Vidal E. (2017). BA71∆CD2: A New Recombinant Live Attenuated African Swine Fever Virus with Cross-Protective Capabilities. J. Virol..

[9] Chen W., Zhao D., He X., Liu R., Wang Z., Zhang X., Li F., Shan D., Chen H., Zhang J. (2020). A seven-gene-deleted African swine fever virus is safe and effective as a live attenuated vaccine in pigs. Sci. China Life Sci..

[10] Zhang Y., Ke J., Zhang J., Yang J., Yue H., Zhou X., Qi Y., Zhu R., Miao F., Li Q. (2021). African Swine Fever Virus Bearing an I226R Gene Deletion Elicits Robust Immunity in Pigs to African Swine Fever. J. Virol..

[11] Zhang J., Zhang Y., Chen T., Yang J., Yue H., Wang L., Zhou X., Qi Y., Han X., Ke J. (2021). Deletion of the L7L-L11L Genes Attenuates ASFV and Induces Protection against Homologous Challenge. Viruses.

[12] Reis A.L., Abrams C.C., Goatley L.C., Netherton C., Chapman D.G., Sanchez-Cordon P., Dixon L.K. (2016). Deletion of African swine fever virus interferon inhibitors from the genome of a virulent isolate reduces virulence in domestic pigs and induces a protective response. Vaccine.

[13] Fan J., Zhu R., Li N., Yang J., Yue H., Zhang Y., Zhou X., Ke J., Wang Y., Li Q. (2023). African swine fever virus I196L is a virulence determinant and its deletant induces robust protection in Domestic pig. bioRxiv.

[14] Camacho C., Coulouris G., Avagyan V., Ma N. (2009). Papadopoulos J, Bealer K, Madden TL. BLAST+: Architecture and applications. BMC Bioinform..

[15] Kelley L.A., Mezulis S., Yates C.M., Wass M.N., Sternberg M.J. (2015). The Phyre2 web portal for protein modeling, prediction and analysis. Nat. Protoc..

[16] Dinhobl M., Spinard E., Tesler N., Birtley H., Signore A., Ambagala A., Masembe C., Borca M.V., Gladue D.P. (2023). Reclassification of ASFV into 7 Biotypes Using Unsupervised Machine Learning. Viruses.

[17] Käll L., Krogh A., Sonnhammer E.L. (2007). Advantages of combined transmembrane topology and signal peptide prediction--the Phobius web server. Nucleic Acids Res..

[18] Letunic I., Khedkar S., Bork P. (2021). SMART: Recent updates, new developments and status in 2020. Nucleic Acids Res..

[19] Spinard E., Azzinaro P., Rai A., Espinoza N., Ramirez-Medina E., Valladares A., Borca M.V., Gladue D.P. (2022). Complete Structural Predictions of the Proteome of African Swine Fever Virus Strain Georgia 2007. Microbiol. Resour. Announc..

[20] Abramson J., Adler J., Dunger J., Evans R., Green T., Pritzel A., Ronneberger O., Willmore L., Ballard A.J., Bambrick J. (2024). Accurate structure prediction of biomolecular interactions with AlphaFold 3. Nature.

[21] Lewis T., Zsak L., Burrage T.G., Lu Z., Kutish G.F., Neilan J.G., Rock D.L. (2000). An African swine fever virus ERV1-ALR homologue, 9GL, affects virion maturation and viral growth in macrophages and viral virulence in swine. J. Virol..

[22] Borca M.V., Carrillo C., Zsak L., Laegreid W.W., Kutish G.F., Neilan J.G., Burrage T.G., Rock D.L. (1998). Deletion of a CD2-like gene, 8-DR, from African swine fever virus affects viral infection in domestic swine. J. Virol..

[23] Borca M.V., O’Donnell V., Holinka L.G., Risatti G.R., Ramirez-Medina E., Vuono E.A., Shi J., Pruitt S., Rai A., Silva E. (2020). Deletion of CD2-like gene from the genome of African swine fever virus strain Georgia does not attenuate virulence in swine. Sci. Rep..

[24] Zsak L., Caler E., Lu Z., Kutish G.F., Neilan J.G., Rock D.L. (1998). A nonessential African swine fever virus gene UK is a significant virulence determinant in domestic swine. J. Virol..

[25] Ramirez-Medina E., Vuono E., O’Donnell V., Holinka L.G., Silva E., Rai A., Pruitt S., Carrillo C., Gladue D.P., Borca M.V. (2019). Differential Effect of the Deletion of African Swine Fever Virus Virulence-Associated Genes in the Induction of Attenuation of theHighly Virulent Georgia Strain. Viruses.

[26] Zsak L., Lu Z., Kutish G.F., Neilan J.G., Rock D.L. (1996). An African swine fever virus virulence-associated gene nl-s with similarity to the herpes simplex virus icp34.5 gene. J. Virol..

[27] Sanford B., Holinka L.G., O’Donnell V., Krug P.W., Carlson J., Alfano M., Carrillo C., Wu P., Lowe A., Risatti G.R. (2016). Deletion of the thymidine kinase gene induces complete attenuation of the Georgia isolate of African swine fever virus. Virus Res..

[28] Moore D.M., Zsak L., Neilan J.G., Lu Z., Rock D.L. (1998). The African swine fever virus thymidine kinase gene is required for efficient replication in swine macrophages and for virulence in swine. J. Virol..

[29] Reis A.L., Goatley L.C., Jabbar T., Sanchez-Cordon P.J., Netherton C.L., Chapman D.A.G., Dixon L.K. (2017). Deletion of the African Swine Fever Virus Gene DP148R Does Not Reduce Virus Replication in Culture but Reduces Virus Virulence in Pigs and Induces High Levels of Protection against Challenge. J. Virol..

[30] Rathakrishnan A., Reis A.L., Goatley L.C., Moffat K., Dixon L.K. (2021). Deletion of the K145R and DP148R Genes from the Virulent ASFV Georgia 2007/1 Isolate Delays the Onset, but Does Not Reduce Severity, of Clinical Signs in Infected Pigs. Viruses.

[31] Ran Y., Li D., Xiong M.G., Liu H.N., Feng T., Shi Z.W., Li Y.H., Wu H.N., Wang S.Y., Zheng H.X. (2022). African swine fever virus I267L acts as an important virulence factor by inhibiting RNA polymerase III-RIG-I-mediated innate immunity. PLoS Pathog..

[32] Zhang Y., Ke J., Zhang J., Yue H., Chen T., Li Q., Zhou X., Qi Y., Zhu R., Wang S. (2021). I267L Is Neither the VirulenceNor the Replication-Related Gene of African Swine Fever Virus and Its Deletant Is an Ideal Fluorescent-Tagged Virulence Strain. Viruses.

